# Exploring the characteristics and potential disparities of non-migrant and migrant colorectal cancer patients regarding their satisfaction and subjective perception of care – a cross-sectional study

**DOI:** 10.1186/s12913-018-3232-5

**Published:** 2018-06-07

**Authors:** Marja Leonhardt, Katja Aschenbrenner, Martin E. Kreis, Johannes C. Lauscher

**Affiliations:** 1 Department of General, Visceral and Vascular Surgery, Campus Benjamin Franklin, Charité-University of Medicine Berlin, 12203 Berlin, Germany; 2 Norwegian National Advisory Unit on Concurrent Substance Abuse and Mental Health Disorders, Innlandet Hospital Trust, Post box 104, 2381 Brumunddal, Norway

**Keywords:** Migrants, Colorectal cancer, Patient satisfaction, Care perception, Questionnaire

## Abstract

**Background:**

Although a fifth of the German population has a migration background, health research regarding this population is scarce. The few existing studies on migrant health show that migrants are faced with restrictions regarding health care due to communication problems, a lack of information and distinct health literacy. Colorectal cancer (CRC) is the second most common tumor disease in Germany. The aim of the study is to explore the potential differences in patient characteristics between migrants and non-migrants with CRC and identify possible disparities between migrants and non-migrants regarding their satisfaction and perception with health care.

**Methods:**

A validated questionnaire was modified for CRC, supplemented with items regarding migration background, translated additionally into Arabic, Turkish and Russian and sent out to 1.694 CRC patients. The outcome indicator was ‘health care satisfaction and experience’ concerning ‘medical consultation’, ‘medical treatment (therapy)’ and ‘hospital stay’ measured on 10-point Likert-scales; explanatory variables were migration background, age, gender, mother tongue, occupation, follow-up care, current discomfort and current treatment. Following descriptive statistics, factor analysis was conducted to compute the outcome variables. Differences between migrants and non-migrants were analyzed using Mann-Whitney-U test and regression analyses.

**Results:**

A total of 522 completed questionnaires – 30.8% response rate – were used for analysis. Patients with a migration background attended less often follow up care than non-migrant patients (74.7% vs. 88.6%; *p* = 0.001). Mean scores regarding satisfaction and experience with consultation, medical treatment (therapy) and hospital stay were 7.86, 7.11 and 7.51 for migrants and 7.84, 7.19 and 7.33 for non-migrants, measured on a 1 to 10 scale with 10 being most satisfied. Migrants were less satisfied with their own involvement in decision making (*p* = 0.029) and the aspect “responsiveness to patient’s questions” (*p* = 0.048) than non-migrants.

**Conclusions:**

Migrants showed less compliance with regard to follow-up care than non-migrants. Furthermore, migrants were more often dissatisfied with communication with the medical staff than non-migrants. This shows the importance of (cross-cultural) communication skills on the part of physicians and nurses.

## Background

Currently, 19.1% of the German population has a migration background. This includes people with a nationality other than German, but also non-migrants whose parents migrated to Germany [1]. People with migration background are a very heterogeneous group in terms of language, cultural and religious background, social situation, health behavior and literacy. Surveys provided evidence that the health of migrants and non-migrants concerning the extent of diseases did not vary that much, except of a few aspects: Typical lifestyle diseases like cardiovascular diseases or cancer, which show a high prevalence in industrial countries, are less frequent within the migrant population. This is explained by healthier dietary habits or the “healthy-migrant-effect” – which is based on the theory that foreign born are in better health than native because only healthy people have the ability to migrate. However, migrants suffer more often from infectious diseases – e.g. the incidence of tuberculosis is five times higher among migrants than among Germans. Furthermore, the smoking prevalence among men and the number of obese women aged 65 years and older is far higher than within the native host-population [2].

Migrants showed, in comparison to Germans, a different behavior regarding use of public health care services. More often than non-migrants, migrants sought help at emergency departments on the weekends and at nighttime, instead of visiting a general practitioner during the regular opening hours. Also the use of prevention services by migrants was below the average of the German population which may be interpreted in terms of communication problems, a lack of information and distinct health literacy [3].

Several studies show that, amongst other factors, survival is a function of ethnicity [4–8].

The latest report of the German Federal Government about morbidity and mortality of migrants concluded that the incidence of gastrointestinal tumors is lower among migrants than in the German host population but was the leading cause of death in the migrant population [9]. More than 60.000 patients are diagnosed with colorectal cancer (CRC) in Germany each year, being the second most common malignancy in Germany. The overall CRC survival rate is 64% [10]. The median age at diagnosis is 71 in men and 75 in women [9] –which equals the age of the first generation migrants.

Migrant health research in Germany is still in its early stages. The present few studies demonstrated that migrants are faced with restrictions regarding health care. Borde et al. showed in a study about the satisfaction of German and Turkish speaking patients with gynecological diseases that the latter are less satisfied with the information given by the physicians as well as the psycho-social and medical care at time of the survey [11]. A positive care experience and thus, the patients’ satisfaction were as essential as the patient-centered care itself. Cancer patient care turned out to be even more important as most of them experience agony, emotional stress and uncertainty [12–14]. Studies have shown that the more satisfied cancer patients were, the more likely they were to cooperate regarding their treatment [15–18]. Patient satisfaction and patient experience were often used synonymously because several factors of experience and perception measure satisfaction [19]. Furthermore, satisfaction studies and patient experience surveys have hardly ever been analyzed regarding migration background. We know to date that there are differences in the health status, health behavior and health care satisfaction [2, 9, 20]. between migrants and non-migrants, but to our knowledge there are no studies comparing patient satisfaction and health care perception between migrants and non-migrants in CRC. However, this information is essential to detect potential deficits in patient care regarding this frequent malignancy and to ensure an ideal and equal health care and therapy for every CRC patient. Hence, the objective of this study was first to explore the characteristics of CRC patients with and without migration background and second to identify possible disparities between migrants and non-migrants regarding their perception of care.

## Methods

### Trial design and participants

A survey with CRC patients (ICD 10 C18-C20: Malignant neoplasm of the colon, the recto-sigmoid junction and the rectum) was conducted between March and August 2015. Patients who were registered between 2004 and 2014 in the prospectively-kept CRC-database of the Charité Comprehensive Cancer Centre (CCCC) and were still alive in 2015 were invited to participate in the study. Age below 18 years was the only exclusion criterion. Of the 1.694 invited patients 687 did not reply and 65 passed away between the last update of the CCCC- database with the national death register (which is only updated every six month) and the mailing of the study-invitations. 117 actively refused to participate, 107 patients signed the informed consent but did not complete the questionnaire, 178 invitations were returned to sender and 540 (31.9%) answered the questionnaire. 18 of these 540 patients were excluded due to insufficient completion of the questionnaire. Thus, data provided by 522 patients was used for the final analysis.

The study was approved by the ethics committee of the medical faculty Charité University Hospital Berlin (EA4/131/14).

### Development and administration of the questionnaire

This study is geared to the patients’ experience definition of Hewitson et al. who described it as “patients’ self-reports of their experience of inpatient care, including staff-patient interactions, information provision, involvement in decisions and support for self-care and overall ratings of care” [21]. A validated questionnaire, which was used in surveys about expectations and perceptions of breast cancer [22]. and ovarian cancer patients [7], was adapted for CRC-patients. To determine whether a person had a migration background, participants were asked for their parents’ place of birth, their citizenship and their mother tongue. The questionnaire was then individually reviewed by experts (1 psychologist, 1 visceral surgeon, 1 study nurse, 1 medical oncologist, 1 social scientist and 1 specialist in internal medicine). A consensus was agreed upon, which was reviewed again by a visceral surgeon and a nurse. The consensus of the adapted questionnaire was pretested on 10 CRC survivors, who were treated at the Charité University Hospital (Campus Benjamin Franklin) due to a condition other than CRC, using an evaluation questionnaire. The results of the pretest (few semantic corrections) were then included in the final version of the German questionnaire, which was translated into Turkish, Arabic and Russian by certified interpreters, to increase the participation rate of non-German native speakers in the survey. The selection of the languages was based on the most frequently spoken languages – beside German – in the city of Berlin [23]. The translations were reviewed by Russian (*n* = 2), Arabic (*n* = 1) and Turkish (*n* = 2) native speakers.

### Measures

#### Outcome indicators “health care perception”

Patients’ health care perception was measured within 18 items – concerning the consultation about the disease, medical treatment (therapy) in terms of knowledge of treating doctors and patient-centered care, success and side effects of systemic and surgical therapy and the hospital stay (facilities, medical and nursing staff, organization and communication). Each item was measured by a 10-point Likert scale (1 being “not satisfied at all/much worse than expected” and 10 being “very much satisfied/much better than expected.”). Based on factor analysis, three components (‘consultation’, ‘medical treatment (therapy)’ and ‘hospital stay’) were identified as outcome indicators that accounted for 67.1% of the variance in the patients’ perception/satisfaction of CRC care. Thus, three sum-scores using the mean of each component were calculated. Patients with missing values on more than half of the items in a scale were excluded. Cronbach’s alpha was 0.71 which is acceptable.

#### Explanatory variables

The variable ‘migration background’ was constructed according to the definition of the Federal Statistic Office of Germany [1]. Here, the migration status is determined by both the birthplace of the patient and the parents. If all three were born in another country other than Germany, the patient is categorised as ‘first-generation migrant’. If the patient, but not the mother and/or the father were born in the country of residence, they were categorised as ‘second-generation’ migrants. Differences between the native population and the migrants (first- and second-generation) were analysed. As age, gender, health status and outcome have been found to affect the satisfaction of patients [24]., the analysis was also controlled for the variables ‘current discomfort’ (no/yes), ‘current treatment’ (no/yes), `compliance to follow-up care’ (no/yes), ‘age’(in years), ‘gender’(male/female) and ‘UICC stage’(UICC 0-II/ III and IV) [25, 26].. Furthermore, the ability to communicate in German [27]. and the employment status of migrants have been interpreted as potential entry barriers to the German healthcare system. Therefore the variables ‘spoken language’ (German or bilingual/other than German) and ‘occupation’ (unemployed, non-academic employee, academic-employee, retired) were included as explanatory variables.

#### Statistical analyses

The sample was characterized using descriptive statistics. For the purpose of analysis, responses in multiple choice questions were dichotomised [7]. Differences between migrants and non-migrants were calculated by the chi-square- or t-test for categorical variables. To show the strength of the relationship, Cramer’s V and the contingency coefficient (CC) were reported. The comparison of migrants and non-migrants regarding the perception/satisfaction of/ with health care was analysed with the nonparametric Mann–Whitney U-Test for each single variable. Additionally, the average median of the variables assessing perception/satisfaction was used as orientation to divide the responses of the 10-point Likert-scale into two answer-categories, scoring 1–7 and 8–10 which were analysed by chi-square-test. Associations between the different aspects of patients’ perception/satisfaction were evaluated by the Pearson’s correlation coefficient and analysed regarding migration background. The three sum-scores, which were computed based on factor analysis, were also used for multivariate regression analysis (ordinary least squares; listwise deletion) to assess whether the perception/satisfaction of CRC care was significantly related to variables such as migration background, age, gender, spoken language, occupation, UICC stage, current treatment, current discomfort and compliance to follow-up care. For the purpose of regression the explanatory variables were computed into dummy variables. Before analysis, the existence of multicollinearity between the control variables was ruled out. No indication of multicollinearity was found regarding the tolerance (> 0.25) and the variance inflation factor (< 10). It was examined whether the migration background has a moderating effect on the patients’ perception/satisfaction concerning the three components ‘consultation’, ‘medical treatment (therapy)’ and ‘hospital stay’ by fitting interaction terms between ‘migration background’ (no/yes) and ‘current discomfort’(no/yes), current treatment (no/yes), UICC stage (0-II/III-IV), compliance to follow-up care (no/yes), age and gender (male/female) in the regression models [28]. The level for statistical significance was set at *p* ≤ 0.05. All tests were conducted in IBM Corp. Released 2016. IBM SPSS Statistics for Windows, Version 24.0. Armonk, NY.

## Results

### Characteristics of the study population

The questionnaires were answered 507 times in German, 9 times in Russian, 4 times in Turkish and 2 times in Arabic. 85 patients (16.3%) had a migration background whereof 61 (71.8%) were first generation migrants and 24 (28.2%) were second generation migrants. The patients with migration background originated from 34 different countries. Altogether, spoken language was strongly associated with the patients’ or their parents’ country of origin (see Table 1 test statistics). The majority of the patients were retired by time of diagnosis (67.1% within the German population, 56.2% within the migrant population) and about a fifth was a non-academic employee (18.3% of the non-migrants; 23.3% of the migrants). Within the migrant study population there were more unemployed (15.0%) and less (5.5%) academic employees than in the host population (4% unemployed; 10.6% academic employees). Chi^2^-test showed an association between migration background and ‘occupation’ Chi^2^(1) = 16.581, (*p* = 0.001, Cramer’s V = 0.192, CC = 0.189). At time of diagnosis, 46 patients (54.8%) with migration background and 240 German patients (55.3%) were diagnosed with an UICC stage 0 to II and 38 (45.2%) patients with a migration background and 193 (44.7%) non-migrants with an UICC stage III or IV (*p* = 0.911). No difference regarding current discomfort (*p* = 0.821) and current treatment (*p* = 0.294) were found between migrants and non-migrants. 25.3% of the migrant patients did not comply with follow-up vs. 11.4% of the Non-migrants. Chi^2^-test showed an association between migration background and ‘compliance to follow up care’ (Chi^2^ (1) = 10.937, *p* = 0.001, Cramer’s V = 0.150, CC = 0.149). Migrants tend to attend less frequently the follow up care than non-migrants. The characteristics of the participating patients are shown in Table 1.

**Table 1:** Characteristic of the study population (total sample: *n* = 522)

	Non-migration background		Migration background	
Mean Age^a^ ± SD	63.84 (11.66)		60.21 (11.68)	
	n	%	n	%
Gende^b^	n total = 437		n total = 85	
Male	252	57.7	54	63.5
Female	185	42.3	31	36.5
Mother tongue^c^:	n total = 437		n total = 85	
German	463	99.5	28	33
Bilingual	0	0	32	37.6
Other than German	2	0.5	25	29.4
Occupation^d^:	n total = 377		n total = 73	
Unemployed	15	4.0	11	15
Non-academic employee	69	18.3	17	23.3
Academic employee	40	10.6	4	5.5
Retired	253	67.1	41	56.2
UICC stage^e^:	n total 433		n total = 84	
UICC 0	9	2.1	4	4.8
UICC I	119	27.5	28	33.3
UICC II	112	25.9	14	16.7
UICC III	108	24.9	29	34.5
UICC IV	85	19.6	9	10.7
In a current treatment*^f^:	n total 436			
Radiotherapy	15	3.4	2	2.4
Chemotherapy	58	13.3	6	7.1
Surgery	17	3.9	6	7.1
Other therapy^1^	119	27.3	21	24.7
Actual current discomfort*^g^:	n total 435			
Nausea/vomiting	16	3.7	8	9.4
Weakness	80	18.4	24	28.2
Numbness/tingling	85	19.5	14	16.5
Pain	53	12.2	16	18.8
Dyspnoea	51	11.7	11	12.9
Skin anomaly	38	8.7	5	5.9
Compliance to follow up care^h^:	n total 405		n total 79	
Yes	359	88.6	59	74.7
No	46	11.4	20	25.3

*n* = number (varies due to missing values); *UICC* = Union for International Cancer Control; *multiple answers possible, ^1^not further defined in the questionnaire

^a^t(520) = 2.620, *p* = 0.009

^b^Chi^2^(1) = 1.009,*p* = 0.315, Cramer’s V = 0.44, CC = 0.44

^c^Chi^2^(1) = 314.848, *p* < 0.001, Cramer’s V = 0.777, CC = 0.613. For the purpose of analysis answer options were dichotomized (German / other or bilingual)

^d^Chi^2^(1) = 16.581, *p* = 0.001, Cramer’s V = 0.192, CC = 0.189

^e^Chi^2^(1) = 0.013, *p* = 0.911, Cramer’s V = 0.005, CC = 0.005. For the purpose of analysis answer options were dichotomized (UICC 0-II/III-IV)

^f^Chi^2^(1) = 1.103, *p* = 0.294, Cramer’s V = 0.046, CC = 0.06. For the purpose of analysis answer options were dichotomized (yes/no)

^g^Chi^2^(1) = 0.051, *p* = 0.821, Cramer’s V = 0.010, CC = 0.010. For the purpose of analysis answer options were dichotomized (yes/no)

^h^Chi^2^(1) = 10.937, *p* = 0.001, Cramer’s V = 0.150, CC = 0.149

By bivariate analysis (Table 2) and splitting the data regarding migration background the different aspects of patients’ perception/satisfaction significantly correlated with each other: the more satisfied they were with their consultation (*p* < 0.001, *r* = 0.353) the more positively they rated their medical treatment (*p* = 0.003, *r* = 0.325) and the more satisfied they were with the hospital stay (*p* < 0.001, *r* = 0.617). These correlations also apply to the group of non-migrants.

**Table 2:** Correlations between aspects of perception/satisfaction - reported Pearson’s Correlation Coefficient

	Non-migrants		Migration background	
	With medical treatment	With hospital stay	With medical treatment	With hospital stay
With consultation	0.374*	0.524*	0.325*	0.617*
With medical treatment		0.418*		0.572*

*p<0.01

### Care perception and satisfaction

On a 10-point-Likert-scale with 1 “not satisfied at all/much worse than expected” and 10 being “very much satisfied/much better than expected”, the mean score of the total study group was 7.84 relating to the aspect of ‘medical consultation’, 7.17 referring to ‘perception of the medical treatment (therapy)’ and 7.36 for the aspect ‘satisfaction with the hospital stay’. In the subgroup of migrants, the mean scores were 7.86, 7.11 and 7.51 vs. 7.84, 7.19 and 7.33 for the group of non-migrants. There were no significant differences between migrants and non-migrants. An overview of the results according to the migration background is visualized in Table 3. Both the migration study population and the German study population were satisfied with the success of the medical treatment/therapy (67.5 and 75.9%). Significant differences between the migration and non-migration population could be found regarding the aspects “responsiveness to patients questions” (Chi^2^ (1) = 3.908, *p* = 0.048) and “own participation in decision making” (Chi^2^ (1) = 4.771, *p* = 0.029). With both, patients with a migration background were less satisfied than non-migrants.

**Table 3:** Care experience and satisfaction

Question	Non-migration background					Migration background				
	N valid	MD	MN	% Scores 1–7	% Scores 8–10	N valid	MD	MN	% Scores 1–7	% Scores 8–10
How satisfied are you with the consultation about your disease regarding…										
elaborateness	430	8	7.70	33.0	67.0	84	8	7.80	34.5	65.5
quality	426	8	7.82	30.0	70.0	81	9	8.04	29.6	70.4
comprehensibility	427	9	8.00	28.1	71.9	83	9	8.05	30.1	69.9
responsiveness to your questions*	424	9	8.08	25.2	74.8	84	9	7.83	35.7	64.3
received information about your disease	422	8	7.55	34.1	65.9	83	9	7.78	36.1	63.9
How satisfied are you with the doctoral treatment concerning…										
the expertise of the physicians	422	9	8.84	14.7	85.3	82	10	8.93	17.1	82.9
my own participation in decision making*	397	8	7.67	32.7	67.3	79	8	7.19	45.6	54.4
the involvement of relatives	397	8	7.08	40.3	59.7	78	8	7.23	41.0	59.0
How would you evaluate…										
the success of your therapy	394	9	8.22	24.1	75.9	80	9	8.18	32.5	67.5
the side-effects	401	8	6.85	46.4	53.6	83	8	6.89	44.6	55.4
the burden due to surgery	420	8	7.48	48.6	51.4	83	8	6.75	49.4	50.6
the pain due to therapy	420	8	7.66	36.9	63.1	84	8	7.15	40.5	59.5
the nausea due to therapy	400	9	6.16	33.8	66.3	83	8	7.19	44.6	55.4
the fatigue due to therapy	418	7	6.90	60.0	40.0	82	7	6.48	54.9	45.1
How satisfied are you with the hospital stay concerning…										
the facilities (e.g. beds, food, medical equipment etc.)	425	8	6.90	47.8	52.2	84	8	7.31	36.9	63.1
the organization and procedures	423	8	7.13	44.2	55.8	84	8	7.24	38.1	61.9
the nursing staff	426	9	7.88	30.3	69.7	84	8	8.15	23.8	76.2
the possibilities to communicate	423	8	7.45	38.3	61.7	84	8	7.36	36.9	63.1

*MD* = median, *MN* = mean, *N valid* = Number of valid cases, Scores: Questions had a 1–10 response scale with 1 “being not satisfied at all/much worse than expected” and 10 being “very much satisfied/much better than expected.” *p < 0.05

Mann-Whitney-U-Test showed no significant differences between migrants and non-migrants in terms of the perceptions and satisfaction regarding the three components ‘consultation’ (U = 17,886.5, *p* = 0.889), ‘medical treatment (therapy)’ (U = 17,519.5, *p* = 0.841) and ‘hospital stay’ (U = 16,945.5, *p* = 0.424).

### Regression analysis

Table 4 shows the results of multivariate regression analysis with the explanatory variables and the three dependent variables experience/satisfaction with the consultation, medical treatment and hospital stay. The explained variance for the models ranged from 8.3 to 14.9%. Regression analysis showed that the ‘migration background’ had no significant association with the patients’ perception/satisfaction (1st generation: *p* = 0.517, 0.807, 0.060; 2nd generation: 0.476, 0.221, 0.688). The following explanatory variables had a significant association with the dependent variables: age was significantly associated with perception/satisfaction of/with the hospital stay and perception/satisfaction of/with the medical treatment (*p* < 0.001, the older the patients the more satisfied they were), while current discomfort was significantly related to the perception of consultation (*p* = 0.001, the more discomfort the less positive perception of consultation) and perception/satisfaction of/with the medical treatment (p < 0.001, the more discomfort the less satisfied they are with the medical treatment). The compliance to follow-up care was associated with the perception/satisfaction of/with consultation (p < 0.001, if patients attend follow up care they are more satisfied with the consultation). The negative unstandardized coefficient represents a negative relationship between the perceptions of/with the consultation and current discomfort: suffering from a discomfort reduces the evaluation of the perception/satisfaction of/with the consultation by 0.7 units (on a scale from 1 to 10) if everything else remain equal.

**Table 4:** Multivariate linear regression models: association between explanatory variables and three dependent variables (perception/satisfaction)

	With consultation		With medical treatment		With hospital Stay	
	*n*=402 Durbin- Watson= 1.753		*n*=396 Durbin- Watson= 1.853		*n*=402 Durbin- Watson=1.591	
	B	*p*	B	*p*	B	*p*
Age	0.013	0.246	0.047	0.000	0.046	0.000
Female (reference male)	-0.007	0.972	-0.044	0.822	-0.143	0.499
Migrant-background (reference: German)						
1^st^ generation	0.469	0.517	-0.169	0.807	1.403	0.060
2^nd^ generation	0.312	0.476	-0.542	0.221	-0.191	0.688
Mother tongue (reference: German)						
Bilingual/other than German	-0.090	0.900	0.390	0.571	-0.807	0.276
Occupation (reference: retired)						
Unemployed	0.048	0.922	0.343	0.496	0.731	0.170
Non-academic employee	0.317	0.310	0.373	0.240	0.499	0.134
Academic-employee	0.803	0.030	0.417	0.269	0.401	0.324
UICC stage (reference: status 0-II)						
III-IV	-0.194	0.329	-0.090	0.465	-0.228	0.293
Compliance to follow-up care (reference: no follow-up care)	1.159	0.000	0.293	0.314	0.571	0.067
Current discomfort (reference: no discomfort)	-0.700	0.001	-1.061	0.000	-0.393	0.078
Current treatment (reference: no treatment)	0.079	0.698	-0.350	0.092	-0.140	0.528

Explained Variance (R^2^): Experience with consultation (0.089), Experience with medical treatment (therapy) (0.149), Experience with hospital stay (0.083)

*B* = regression coefficient, *n*= number of study patients

### Moderating effect

To test the hypothesis that the relationship between the perception of/with ‘consultation’, ‘medical treatment’ or ‘hospital stay’ and currently suffering a discomfort was different according to the migration background, an interaction term (migration background X current discomfort) was added to the regression model. Current discomfort had a negative influence on how positively patients rated their perception of the consultation (*p* = 0.024), but had no significant influence on satisfaction with the medical treatment or hospital stay However, the effects were stronger within the group of non-migrants compared to the group of migrants. Regression models with the three components of patient perception/satisfaction as dependent variables were also computed with further interaction terms (migration background X the variables ‘gender’ or occupation or ‘UICC-stage’ or ‘current treatment’ or ‘follow-up-care’) but none of them show any significance according to the interaction term.

## Discussion

This study explores the characteristics of CRC patients and potential disparities regarding their satisfaction and subjective perception of care. The study did not find differences between migrants and non-migrants regarding their care perceptions and satisfaction of/with the medical consultation, treatment (therapy) and hospital stay. But, looking at the solitary variables of the three superordinate components of perception and satisfaction, patients with a migration background were less satisfied with some aspects of communication with the medical staff. Only in two single aspects of medical consultation, that is participation in decision making and responsiveness to questions, patients with a migration background were less satisfied with aspects of communication with the medical staff.

With regards to characteristics of the migrant and non-migrant study population, we found a higher rate of unemployed patients in the group of migrants and a higher rate of non-compliance to follow-up in this group compared to non-migrants. To our knowledge, the lower compliance for the migrant population in Germany in follow-up care has not been described before and deserves further attention. Further studies and efforts should focus on methods to increase adherence to follow-up in migrants diagnosed with colorectal cancer because a survival benefit for adequate follow-up in CRC has been shown [29].

Patients with and without a migration background do not differ in terms of gender and age distribution, UICC-stage by time of diagnosis, suffering from current discomfort or receiving current treatment. Patients with a migration background were more often unemployed or in a non-academic occupation than German patients. This is in line with the fact that people with a migration background more often tend to carry out poorly paid jobs and are less qualified than non-migrants [30]. Furthermore, migrants less often attend prevention care [2], which may explain the lower rate of compliance to follow-up care among migrants compared to the German host population.

Three components of patient perception and satisfaction of/with colorectal cancer related health care could be identified within the study: components related (1) to the consultation, (2) to the medical treatment (therapy) and (3) to the hospital stay. Overall, patients – regardless of their migration background – had a very positive care perception. The outcome of our study shows that a complete medical consultation has a positive influence on the patients’ satisfaction with the medical treatment and the hospital stay. Assuming that a well-informed patient has good health literacy the latter has been found to influence the health related quality of life in a positive manner [31]. This emphasizes the importance of all aspects playing a role in the cancer consultation such as the quality and comprehensibility as well as the involvement of the patient himself into decisions regarding the therapy and treatment. A good consultation implies a good patient-physician relationship and this again requires empathy for the counterpart and – in case of a migrant patient – cross-cultural competence. Therefore it should be considered to implement cross-cultural communication as an obligatory module within the medical education. Cultural knowledge and sensitive communication matched with the avoidance of stereotyping and tolerance on behalf of the medical staff may avoid cultural communication barriers. Likewise, there is a minority who is not satisfied with their own participation in decision-making and the responsiveness to their questions. In the latter two, the percentage within the group of migrants is higher than in non-migrants. While the majority of migrants were satisfied with their participation in decision-making and the possibilities to communicate, a portion of the migrant population found these aspects to be suboptimal. A study in Australia showed that cancer patients with migration background experienced passive involvement during treatment consultations [32]. This demonstrates once more that (cross-cultural) communication is an important issue in care and that there is room for improvement in communication with migrants and their involvement in decision-making.

On the other hand, we did not find differences in terms of the perceptions and satisfaction regarding the three superordinate components ‘consultation’, ‘medical treatment (therapy)’ and ‘hospital stay’ between non-migrants and migrants in our study. This is in line with the study of Kietzmann et al [33]. which found that migration status per se did not show any influence regarding the overall satisfaction with pre-hospital emergency care. Our results could also be explained by the ‘social integration hypothesis’ [34, 35], which is based on the theory that the more contact between migrants and the host population takes place the more the migrants become integrated. The more migrants – with 1st generation migrants leading the way – have contact with non-migrants the more they are satisfied with their health care. In the present study the majority of patients with a migration background belonged to the 1st generation migrants and stated German as the language in which they may express themselves best. This implies that the majority of participating migrants already had spent some years in Germany and got the chance to get adapted to the host society.

The fact that patients who suffer from current discomfort are less satisfied with their consultation may be interpreted in terms of the circumstance that health problems generate negative emotions. These are attributed to the care provider and thus the consultation. That current discomfort has a negative influence on the patients’ satisfaction corresponds to the results of another study which examined factors that influence cancer patients’ overall perception of the quality of care [36]. What is more, the results of the regression analyses in the present study have to be interpreted with caution as the explained variance of the three regression models – due to the characteristics of the outcome variables which assess human behaviour – is low, but comparable to other studies dealing with patient satisfaction with care [17, 33]. Since the results were both statistical and clinical significant we account the model as suitable.

Several study limitations should be noted. The overall response rate of 31.9% is fairly low. We know that 3.8% of the study population died between database-clearing in 2014 and conduction of the survey in 2015. Considering that 45% of the patients were UICC-stage 3 and -stage 4, we can assume that about half of the non-responders (40.6% no response and 10.5% invitations back to sender) died due to the severity of the disease, which does explain our low response rate. Due to the small sample of patients with migration background (16.3%) and its heterogeneity (migrants originated from 34 different countries), subgroup analysis – regarding 1st generation and 2nd generation migrants or the countries of origin – was omitted as the results would be skewed. Furthermore, people with migration background have to overcome language and cultural barriers in order to participate in a trial [37]. To reduce the language barrier, our study material was translated into Arabic, Turkish and Russian but only a few study participants made use of the translated versions. On the other hand there is little missing data in the existing questionnaires so we assume that the majority of the responders understood the study material – even in German – as most of them stated that German is the language in which they can express themselves best. Another reason for the low response rate of patients with migration background can be the low level of literacy and awareness of medical research [38]. According to the German Federal Office for Migration and Refugees, more people with migration background lack educational attainment compared to non-migrants [39]. Assuming that people with low education have little knowledge of the importance of clinical research could be another potential explanation for the low response rate of migrants. There might be bias due to the fact that more educated migrants with higher language skills were more likely to answer the questionnaire. The fact that the majority rated their perception/satisfaction of/with the consultation, medical treatment and hospital stay as very positive may have been caused by social desirability bias – a tendency to under- or over report something in order to avoid being viewed negatively by others [40]. Although questions assessing the care perception did not touch very sensitive topics, they might have pressured the participants to report more positive scores as most of them were still in a follow-up care program by time if the survey. Johnson and van der Vijver [41]. found out that social exclusion, discrimination and less political rights of migrants in their host country may lead to the tendency that minorities give high values within surveys. Although our questionnaire was pretested, social desirability and acquiescence bias cannot be avoided entirely as the migrant population is very heterogeneous.

Given the growing number of migrants in Europe and worldwide and the identification of differences in health care utilization between migrants and non-migrants, these topics have gained more interest in the media as well as in scientific research. Although colorectal carcinoma is one of the most common cancers worldwide, data regarding the evaluation of satisfaction with health care of migrants and non-migrants in colorectal cancer is very scarce.

Despite the limitations outlined above, this study is to our knowledge the largest study addressing the evaluation of consultation, medical treatment and hospital stay of migrants and non-migrants diagnosed with colorectal cancer.

## Conclusions

In summary, no major disparities between migrant and non-migrant patients were found when it comes to the overall satisfaction and perception with/of the medical consultation, medical treatment and hospital stay. However, patients with a migration background showed less compliance with regards to follow-up care than non-migrants and were less satisfied with aspects of communication. Although the number of migrants is small compared to the complete study population, these results should be taken into account within further research. Second, these findings support that an all-encompassing medical consultation has a positive influence on patients’ satisfaction with medical treatment and hospital stay – regardless of their ethnical background. This underlines the importance of good (cross-cultural) communication skills on the part of the medical staff.

## Abbreviations

CC: Contingency Coefficient
CCCC: Charité Comprehensive Cancer Center
CRC: Colorectal cancer
ICD: International Classification of Diseases
TNM: Classification of Malignant Tumors (T = tumor, N = lymph nodes, M = metastasis)
UICC: *Union Internationale contre le cancer*
